# Incidence of Nosocomial COVID-19 in Patients Hospitalized at a Large US Academic Medical Center

**DOI:** 10.1001/jamanetworkopen.2020.20498

**Published:** 2020-09-09

**Authors:** Chanu Rhee, Meghan Baker, Vineeta Vaidya, Robert Tucker, Andrew Resnick, Charles A. Morris, Michael Klompas

**Affiliations:** 1 Department of Population Medicine, Harvard Medical School and Harvard Pilgrim Health Care Institute, Boston, Massachusetts; 2 Division of Infectious Diseases, Brigham and Women’s Hospital, Boston, Massachusetts; 3 Infection Control Department, Brigham and Women’s Hospital, Boston, Massachusetts; 4 Department of Quality and Safety, Brigham and Women’s Hospital, Boston, Massachusetts; 5 Department of Medicine, Brigham and Women’s Hospital, Boston, Massachusetts

## Abstract

**Importance:**

Some patients are avoiding essential care for fear of contracting coronavirus disease 2019 (COVID-19) in hospitals. There are few data, however, on the risk of acquiring COVID-19 in US hospitals.

**Objective:**

To assess the incidence of COVID-19 among patients hospitalized at a large US academic medical center in the 12 weeks after the first inpatient case was identified.

**Design, Setting, and Participants:**

This cohort study included all patients admitted to Brigham and Women’s Hospital (Boston, Massachusetts) between March 7 and May 30, 2020. Follow-up occurred through June 17, 2020. Medical records for all patients who first tested positive for severe acute respiratory syndrome coronavirus 2 (SARS-CoV-2) by reverse-transcription polymerase chain reaction (RT-PCR) on hospital day 3 or later or within 14 days of discharge were reviewed.

**Exposures:**

A comprehensive infection control program was implemented that included dedicated COVID-19 units with airborne infection isolation rooms, personal protective equipment in accordance with US Centers for Disease Control and Prevention recommendations, personal protective equipment donning and doffing monitors, universal masking, restriction of visitors, and liberal RT-PCR testing of symptomatic and asymptomatic patients.

**Main Outcomes and Measures:**

Whether infection was community or hospital acquired based on timing of tests, clinical course, and exposures.

**Results:**

Over the 12-week period, 9149 patients (mean [SD] age, 46.1 [26.4] years; median [IQR] age, 51 years [30-67 years]; 5243 female [57.3%]) were admitted to the hospital, for whom 7394 SARS-CoV-2 RT-PCR tests were performed; 697 COVID-19 cases were confirmed, translating into 8656 days of COVID-19–related care. Twelve of the 697 hospitalized patients with COVID-19 (1.7%) first tested positive on hospital day 3 or later (median, 4 days; range, 3-15 days). Of these, only 1 case was deemed to be hospital acquired, most likely from a presymptomatic spouse who was visiting daily and diagnosed with COVID-19 before visitor restrictions and masking were implemented. Among 8370 patients with non–COVID-19–related hospitalizations discharged through June 17, 11 (0.1%) tested positive within 14 days (median time to diagnosis, 6 days; range, 1-14 days). Only 1 case was deemed likely to be hospital acquired, albeit with no known exposures.

**Conclusions and Relevance:**

In this cohort study of patients in a large academic medical center with rigorous infection control measures, nosocomial COVID-19 was rare during the height of the pandemic in the region. These findings may inform practices in other institutions and provide reassurance to patients concerned about contracting COVID-19 in hospitals.

## Key Points

**Question:**

What is the incidence of hospital-acquired coronavirus disease 2019 (COVID-19) at a large US academic medical center?

**Findings:**

In this cohort study of 9149 patients admitted to a large US academic medical center over a 12-week period, 697 were diagnosed with COVID-19. In the context of a comprehensive and progressive infection control program, only 2 hospital-acquired cases were detected: 1 patient was likely infected by a presymptomatic spouse before visitor restrictions were implemented, and 1 patient developed symptoms 4 days after a 16-day hospitalization but without known exposures in the hospital.

**Meaning:**

These findings suggest that overall risk of hospital-acquired COVID-19 was low and that rigorous infection control measures may be associated with minimized risk.

## Teaser

This cohort study evaluates the incidence of nosocomial coronavirus disease 2019 (COVID-19) among patients admitted to a US medical center in the context of a comprehensive and progressive infection control program.

## Introduction

Many patients have been avoiding essential care during the coronavirus disease 2019 (COVID-19) pandemic owing to fear of contracting severe acute respiratory syndrome coronavirus 2 (SARS-CoV-2) infection in health care settings. This lack of care seeking has been associated with an increase in mortality rates from non-COVID conditions.^1,2,3,4^ Some of this anxiety may have been provoked by reports of widespread outbreaks of SARS-CoV-2 infection within skilled nursing facilities and other congregate settings.^5,6^ There are few data, however, on the adequacy of infection control practices and the risk of acquiring COVID-19 in US acute care hospitals.^7^ Over the first 12 weeks of the pandemic in the region, approximately 700 patients were admitted to Brigham and Women’s Hospital with COVID-19 and more than 8000 without COVID-19. We reviewed all patients diagnosed with COVID-19 on hospital day 3 or later or within 14 days of hospital discharge to quantify the incidence of nosocomial transmission and to assess the effectiveness of the infection control program at the hospital.

## Methods

### Study Design, Setting, and Patient Population

This cohort study included all patients admitted to Brigham and Women’s Hospital, a 793-bed academic medical center in Boston, Massachusetts, from March 7 (when the first patient with COVID-19 was admitted) through May 30, 2020. Study follow-up occurred through June 17. We defined COVID-19–related hospitalizations by a positive SARS-CoV-2 reverse-transcription polymerase chain reaction (RT-PCR) test result during hospitalization or within the 14 days before admission. For patients with COVID-19 who had multiple hospitalizations, only the index hospitalization for COVID-19 was evaluated to determine whether SARS-CoV-2 infection was potentially hospital acquired. The study was deemed exempt from full review by the Mass General Brigham Institutional Review Board owing to its minimal risk and because the study involved only information collected and analyzed as part of routine hospital operations. This study followed the Strengthening the Reporting of Observational Studies in Epidemiology (STROBE) reporting guideline.^8^

We identified all patients with COVID-19 for whom the first positive RT-PCR test result occurred on hospital day 3 or later (with day of admission defined as day 1) or within 14 days after hospital discharge. Two infection control physicians (C.R. and M.K.) independently reviewed the medical records of each case to determine whether SARS-CoV-2 infection was most likely acquired before hospitalization or in the hospital based on timing of symptoms and RT-PCR tests and potential exposures within or outside the hospital (Table 1). Postdischarge cases included patients discharged from the hospital who then tested positive for SARS-CoV-2 anywhere within the Mass General Brigham Healthcare System, which includes 11 acute care hospitals, a rehabilitation hospital network, and numerous outpatient COVID-19 testing facilities within Massachusetts.

**Table 1.** Criteria for Classifying COVID-19 Cases as Community or Hospital Acquired^a^

Classification	Hospitalized patients diagnosed on hospital day 3 or later	Discharged patients diagnosed within 14 d of discharge
Definitely community acquired	Symptoms present on admission and first positive RT-PCR test result on hospital days 3 to 7	Not applicable
Likely community acquired	Symptoms present on admission, first positive RT-PCR test result on hospital days 8 to 14, not tested before day 8 Symptom onset and first positive RT-PCR test result on hospital days 3 to 7 with no known exposures on hospital days 1 and 2 Symptom onset and first positive RT-PCR test result on hospital days 8 to 14, with known exposures or risk factors before hospitalization (within 14 d of symptom onset)	Symptom onset and first positive RT-PCR test result on postdischarge days 8 to 14, known exposures or risk factors outside hospital, and no known exposures during hospitalization Symptom onset and first positive RT-PCR test result on postdischarge days 3 to 7 (or days 1 to 7 if duration of hospitalization was 3 d of less), known exposures or risk factors before hospitalization (for hospital stays of 3 d of less) or after hospitalization, and no known exposures during hospitalization
Likely hospital acquired	Symptom onset and first positive RT-PCR test result on hospital days 3 to 7, known exposures in the hospital on days 1 to 2, and no known exposures or risk factors before hospitalization Symptom onset and first positive RT-PCR test result on hospital days 8 to 14 and no known exposures or risk factors before or during hospitalization	Symptom onset and first positive RT-PCR test result on postdischarge days 1 to 7 after hospital stay of longer than 3 d, no known exposures during hospitalization, and no known exposures or risk factors outside the hospital Symptom onset and first positive RT-PCR test result on postdischarge days 8 to 14, known exposure during hospitalization (within 14 d of symptom onset), and no known exposures or risk factors outside the hospital
Definitely hospital acquired	Symptom onset and first positive RT-PCR test result on hospital day 15 of after	Symptom onset and first positive RT-PCR test result on postdischarge days 1 to 7 with known exposure during hospitalization and no known exposures or risk factors outside the hospital
Unknown	None of the criteria above	None of the criteria above

Abbreviations: COVID-19, coronavirus disease 2019; RT-PCR, reverse-transcription polymerase chain reaction.

^a^ Risk factors included congregate settings, such as rehabilitation or skilled nursing facilities (particularly if there were known COVID-19 outbreaks within the facilities), homelessness or working with homeless persons, and treatment in a hemodialysis center. Patient exposures were defined as cumulative face-to-face time of 10 minutes or longer with a known COVID-positive person when 1 or more person was not wearing a face mask or if the patient shared a room with a COVID-positive roommate.

### Infection Control and Testing Standards

The infection control program evolved during the study period but included screening of all patients for COVID-19 symptoms on admission and daily thereafter, liberal use of RT-PCR testing initially for all symptomatic patients and subsequently for all patients at the time of admission (including asymptomatic patients), dedicated COVID-19 units with airborne infection isolation rooms, personal protective equipment (PPE) in accordance with US Centers for Disease Control and Prevention recommendations, PPE donning and doffing monitors, universal masking of staff and subsequently patients and visitors, and restriction of visitors (Table 2).

**Table 2.** Timeline and Description of Major Infection Control Policies and Interventions

Date	Policy or intervention	Detailed description
Late February	24-7 Personal protective equipment donning and doffing monitors	In the weeks leading up to the first confirmed case of COVID-19, a group of staff members were trained and deployed to observe and assist in PPE donning and doffing for anyone entering the room of a patient with suspected or confirmed COVID-19 by using standardized checklists.
March 13	First COVID-19 ward opened	The first dedicated COVID-19 ward was opened for non–critically ill patients with suspected or confirmed COVID-19. All rooms were set to negative airflow. Standardized protocols were deployed for clinical care, infection control, PPE use, and environmental cleaning. Numerous additional dedicated COVID-19 wards and ICUs were subsequently opened in the ensuing weeks to handle the surge. All staff in these units used N95 respirators or PAPRs, eye protection, gown, and gloves for routine care.
March 18	In-house RT-PCR testing	An in-house laboratory-developed RT-PCR test was developed and went live with a turn-around time of approximately 12 h. Before this, all SARS-CoV-2 RT-PCR tests were sent to the Massachusetts state laboratory, with an mean turn-around time of 2 to 3 d. Additional platforms with faster turn-around times (Hologic Panther Fusion and Cepheid Xpert assays) were subsequently deployed and used for the majority of the study period. The default pathway for ruling out COVID-19 in suspected cases required 2 negative RT-PCR test results at least 12 h apart.
March 25	Universal masking of health care workers	All health care workers were required to wear surgical or procedural masks while on facility premises.
March 28	Mandatory health care worker daily symptom attestation	All health care workers were required to attest online to the absence of any symptoms consistent with COVID-19 before work. Any health care worker with symptoms was not allowed to work and was referred for SARS-CoV-2 RT-PCR testing and occupational health evaluation.
April 3	Restriction of visitors	All visitors were restricted from entering the hospital except under a limited set of circumstances, such as end-of-life care, labor and delivery, or to accompany pediatric and other special care populations.
April 6	Universal masking of visitors and patients	Visitors who were allowed to enter the hospital were required to wear masks at all times. All patients presenting to the emergency department were also masked on arrival. Once roomed in an inpatient unit, patients were allowed to remove masks but were asked to wear them again during encounters with health care workers and outside their rooms.
April 10	Hospital-wide shift to N95 masks for routine COVID-19 care	Outside the dedicated COVID-19 units, staff initially used surgical or procedural masks for respiratory protection for routine care of suspected COVID-19 cases, with N95 masks and PAPRs reserved for aerosol-generating procedures. Once N95 mask supply improved, the PPE standard was modified to N95 masks in all areas of the hospital regardless of need for aerosol-generating procedures, in accordance with CDC guidelines.
April 17	Daily nursing screening for COVID-19 symptoms	A daily nursing screening protocol for possible COVID-19 symptoms was implemented in the electronic health record system. If patients screened positive, a best practice alert was triggered suggesting a discussion with the patient’s physicians to consider RT-PCR testing.
April 26	Universal testing on admission	RT-PCR testing by nasopharyngeal swab was extended to all patients on admission to the hospital regardless of symptoms. Asymptomatic patients required 1 negative test result on admission to the hospital.
May 8	Enhanced eye protection for health care workers	Eye protection was mandated for health care workers caring for any patient who was unable to wear a mask during the encounter, even if the patient tested negative for SARS-CoV-2 on admission.

Abbreviations: CDC, Centers for Disease Control and Prevention; COVID-19, coronavirus disease 2019; ICU, intensive care unit; PAPR, powered air purifying respirators; PPE, personal protective equipment; RT-PCR, reverse-transcription polymerase chain reaction; SARS-CoV-2, severe acute respiratory syndrome coronavirus 2.

The hospital protocol to discontinue isolation precautions for patients with suspected COVID-19 required 2 negative RT-PCR test results from nasopharyngeal swab samples obtained more than 12 hours apart for most patients or 1 negative test result if an alternate diagnosis became apparent. If the patient had a productive cough or required mechanical ventilation, at least 1 of the 2 negative test results had to be from a lower respiratory tract sample. Initially, respiratory samples were sent to the Massachusetts Department of Public Health for RT-PCR testing, but during the majority of the study period, RT-PCR tests were conducted in the laboratory at our hospital on the Hologic Panther Fusion or Cepheid Xpert platforms.

### Statistical Analysis

Descriptive analyses of patient and hospitalization characteristics were conducted using SAS, version 9.4 (SAS Institute).

## Results

Between March 7 and May 30, 2020, 9149 patients (mean [SD] age, 46.1 [26.4] years; median [IQR] age, 51 years [30-67 years]; 5243 female [57.3%]) were admitted to the hospital, for whom 7394 SARS-CoV-2 RT-PCR tests were performed; 697 patients were diagnosed with their first episode of COVID-19. The inpatient COVID-19 census peaked at 171 patients on April 21, 2020. The median length of stay among patients with COVID-19 was 7 days (range, 1-74 days), translating into 8656 days of COVID-19–related care.

### Late-onset COVID-19 Cases in Hospitalized Patients

Of the 697 hospitalized patients with confirmed COVID-19, 12 (1.7%) were first diagnosed on hospital day 3 or later (Figure). The median time from admission to the first positive RT-PCR test result for these 12 patients was 4 days (range, 3-15 days). Their clinical courses are summarized in eTable 1 in the Supplement. None of the 12 patients had known exposures to staff members with COVID-19 or shared rooms with patients with confirmed COVID-19. On medical record review, infection was deemed to be definitely community acquired for 4 patients and likely for 7. Only 1 patient definitely acquired COVID-19 in the hospital because the symptoms began on hospital day 15. This patient was most likely infected by a presymptomatic spouse who was visiting daily until a diagnosis of COVID-19 was made 1 week before the inpatient’s symptoms began. This case occurred before implementation of visitor restrictions and universal masking of all health care workers and patients.

**Figure.**
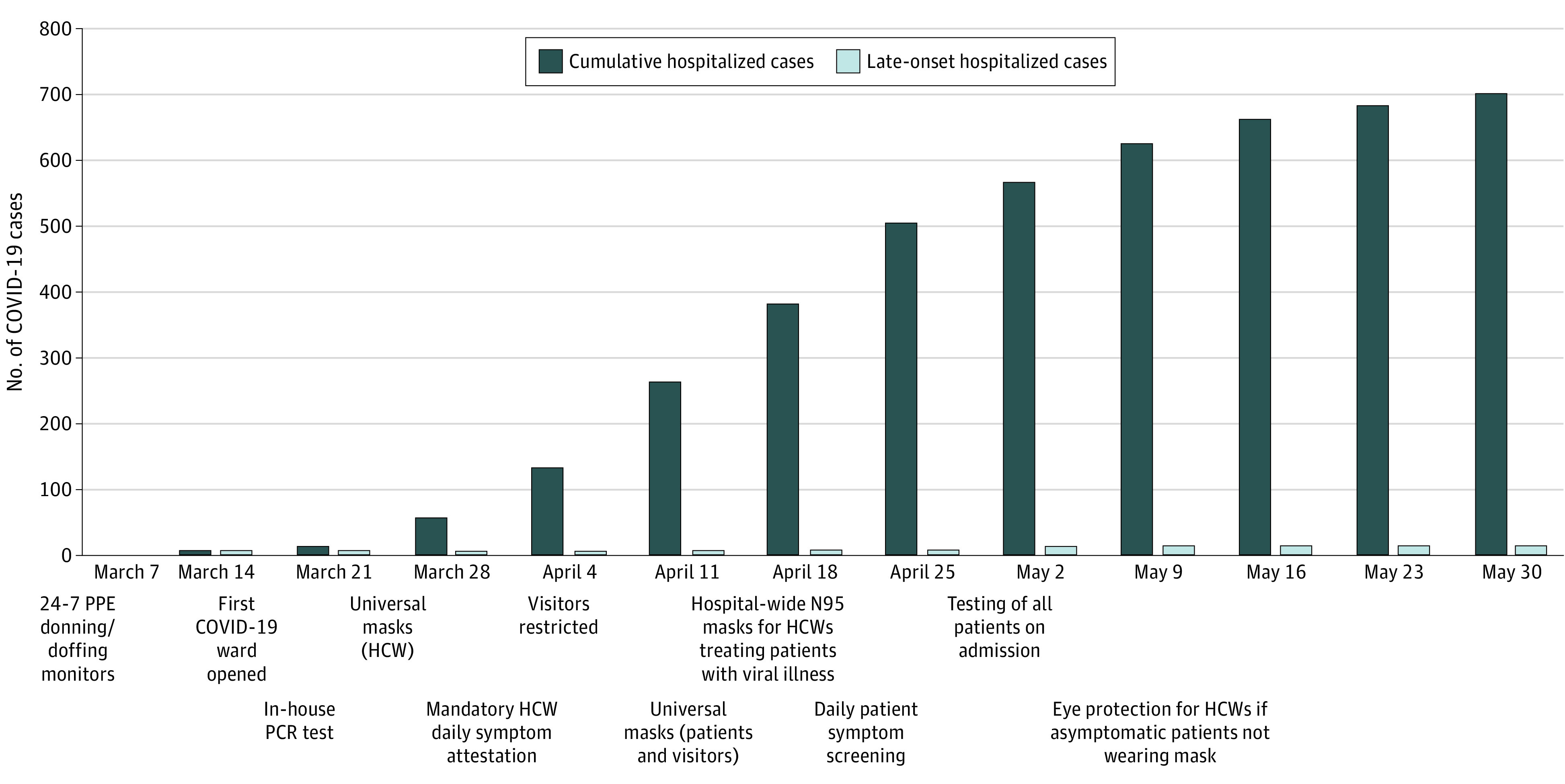
Cumulative Number of Total and Late-Onset Hospitalized Coronavirus Disease 2019 (COVID-19) Cases by Week and Associated With Infection Control Policies Late-onset hospitalized COVID-19 cases were defined as patients who first tested positive for severe acute respiratory syndrome coronavirus 2 by reverse-transcription polymerase chain reaction (PCR) on hospital day 3 or later. Table 2 gives a detailed description of the major infection control policies and interventions. HCW indicates health care worker; PPE, personal protective equipment.

Among the 11 definite or likely community-acquired cases, factors associated with late diagnosis (on or after hospital day 3) included delayed suspicion and testing for COVID-19 because symptoms at admission were attributed to an alternate cause (2 cases); initial negative RT-PCR test results followed by positive test results on serial samples from patients with high suspicion for COVID-19 (3 cases); lack of testing at admission owing to absence of symptoms but with onset of symptoms triggering testing 1 to 2 days later (2 cases); and delayed onset of symptoms in patients with epidemiologic risk factors who tested negative for SARS-CoV-2 on admission when the virus was still in the early incubation period (4 cases). Overall, of the 12 cases first diagnosed on hospital day 3 or later, 9 occurred in the first 7 weeks before universal testing of all patients on admission and 3 occurred in the subsequent 5 weeks after universal testing of all admitted patients was implemented.

### Postdischarge COVID-19 Cases

Among 8370 patients who were hospitalized with non–COVID-19–related conditions and were discharged through June 17, 2020, 11 (0.1%) tested positive in our health care system within 14 days of discharge (median time to diagnosis, 6 days; range 1-14 days) (cases are summarized in eTable 2 in the Supplement). Only 1 case was deemed to be likely hospital acquired, albeit with no known exposures inside the hospital. This patient had a prolonged postoperative hospitalization and developed new febrile symptoms 4 days after discharge, with no known contacts with illness at home or high-risk epidemiologic factors. Two other patients who received diagnoses soon after discharge most likely had delayed diagnoses of COVID-19 because they presented with progression of the same syndromes responsible for their initial hospitalizations but were not tested at the initial admission; these cases occurred in March before more aggressive testing practices were instituted. Another patient most likely had false-negative test results during the initial hospitalization as the patient presented to the hospital again with progression of the same syndrome that had been ongoing for 4 weeks and had COVID-19 diagnosed based on an RT-PCR test result with a borderline cycle threshold value. The remaining 7 cases were likely acquired after discharge: 3 patients had high-risk exposures after discharge, and 4 were discharged to rehabilitation or skilled nursing facilities with COVID-19 outbreaks.

None of the 11 patients diagnosed with COVID-19 after discharge shared a room with a patient with confirmed COVID-19. One patient received care from a staff member diagnosed with COVID-19 but also lived with a spouse who tested positive for SARS-CoV-2 1 week before the patient became ill. This case was considered to likely be community acquired given the high rate of SARS-CoV-2 transmission in households, the patient’s greater contact with the spouse than with the health care worker, and that universal masking of all health care workers had been implemented by that point.

## Discussion

COVID-19 presents important infection control challenges. A substantial proportion of patients are asymptomatic or presymptomatic yet highly contagious.^9,10,11^ Current diagnostic tests are imperfect, especially early in the incubation period, and patients may not develop symptoms until 14 days or more after inoculation.^12^ In addition, although the primary mode of transmission is thought to be through close contact and exposure to droplets, infection from contaminated fomites is possible and the role of airborne transmission remains a subject of debate.^13,14,15^ Our analysis, however, revealed that a multifaceted infection control program based on US Centers for Disease Control and Prevention guidance may be associated with minimized risk of nosocomial transmission of SARS-CoV-2 infection. Over the first 12 weeks of the pandemic in the US, our hospital cared for more than 9000 patients, including approximately 700 with COVID-19 who were present for 8656 hospital-days. Despite the high burden of COVID-19 in our hospital, we identified only 2 patients who likely acquired the infection in the hospital, including 1 who was most likely infected by a spouse before visitor restrictions and universal masking.

The present findings differ from the results of a recent review that suggested that up to 44% of COVID-19 infections may be nosocomial.^16^ However, that review was limited to case series conducted early in the outbreak in Wuhan, China, before recognition of the virus and the institution of infection control practices and PPE. In contrast, hospitals in Hong Kong cared for 42 patients with COVID-19 and reported no nosocomial transmission during the first 6 weeks after the virus was first discovered in China.^17^ Of note, their report encompassed a smaller number of patients compared with the present series.

An important theme that emerged from our case reviews was the need to conduct serial testing of patients with clinical syndromes highly suspicious for COVID-19. At least 3 patients with concerning syndromes initially tested negative for SARS-CoV-2 but had positive results on repeat testing. Other researchers have also documented that repeat RT-PCR testing can yield positive results for patients with initial negative results, albeit at relatively low rates.^18^ On the basis of our early experience, we instituted a protocol requiring at least 2 negative RT-PCR test results for symptomatic patients before discontinuing isolation.

Another observation that emerged was that several patients were only tested for the first time 3 or more days after hospitalization, sometimes because of atypical symptoms that were initially attributed to non-COVID conditions. These cases highlight the importance of implementing universal testing on admission, and we observed fewer late-onset cases after this intervention. However, universal testing is not infallible because several patients initially tested negative while asymptomatic and then tested positive after symptoms began several days later. This underscores the lower sensitivity of RT-PCR early in the course of infection.^19^

### Strengths and Limitations

This study has strengths. This was a comprehensive analysis and review of all patients who first tested positive for SARS-CoV-2 on hospital day 3 or later or within 14 days of hospital discharge. Recent national regulations only require reporting of cases first diagnosed on hospital day 14 or later.^15,20^ Although this strategy ensures that most reported cases were truly acquired in the hospital, it renders hospitals blind to nosocomial infections that manifested before 14 days (which may be common because the mean incubation period for SARS-CoV-2 is 5 days) or after hospitalization.^12^

This study also has limitations. First, it is difficult to know the source of infection in every case. Viral genome sequencing could potentially have helped in some cases, such as for the patient exposed to both a COVID-positive staff member and spouse; however, this was not readily available to us.^21,22^ Even if that case and every other late-onset or postdischarge case were truly hospital acquired, this would represent a low rate of nosocomial infection. Second, despite the implementation of aggressive testing practices, there may have been additional undetected nosocomial cases related to false-negative RT-PCR test results or from patients who might have acquired asymptomatic infection in the hospital but were never tested. Third, there may have been patients who were diagnosed after discharge outside our health care system. However, our health care system includes a broad catchment area. Fourth, we may have missed patients who developed symptoms soon after discharge but did not seek testing until after 14 days. However, we believed that a 14-day surveillance window achieved a reasonable balance between capturing potential hospital-onset cases and avoiding excessive noise from patients who were exposed in the community. Fifth, our findings do not provide insight into the risk of nosocomial infection among health care workers. We believe this warrants a separate detailed analysis. Sixth, it is unclear which of the measures that we implemented were most effective, particularly as our policies evolved rapidly. Some measures, such as the use of airborne infection isolation rooms for all patients with COVID-19 or PPE donning and doffing monitors, are not feasible in all hospitals and may not be necessary to prevent nosocomial transmission. Seventh, there is variation in adherence to basic infection control practices across hospitals, and this may be particularly true during a pandemic because some hospitals have been overwhelmed with patients with COVID-19 while others have remained within capacity. Our hospital cared for many patients with COVID-19 in newly improvised intensive care units and wards but never exceeded surge capacity. As such, our findings may not be generalizable to all hospitals.

## Conclusions

These findings suggest that robust and rigorous infection control practices may be associated with minimized risk of nosocomial spread of COVID-19 to hospitalized patients. These results, especially if replicated at other US hospitals, should provide reassurance to patients as some health care systems reopen services and others continue to face COVID-19 surges.

## Notes

Supplement. **eTable 1.** Summary and Attribution of Infection Acquisition for Late-Onset COVID-19 Cases in Hospitalized Patients **eTable 2.** Summary and Attribution of Infection Acquisition for COVID-19 Cases Diagnosed Within 14 Days of Hospital Discharge
