# Supplementary material for: Incidence of Nosocomial COVID-19 in Patients Hospitalized at a Large US Academic Medical Center
Source: JAMA Netw Open. 2020 Sep 9;3(9):e2020498. doi: 10.1001/jamanetworkopen.2020.20498 (PMC7489854; doi:10.1001/jamanetworkopen.2020.20498)
Supplement: Supplement. — eTable 1. Summary and Attribution of Infection Acquisition for Late-Onset COVID-19 Cases in Hospitalized Patients eTable 2. Summary and Attribution of Infection Acquisition for COVID-19 Cases Diagnosed Within 14 Days of Hospital Discharge [file jamanetwopen-e2020498-s001.pdf]

# Supplementary Online Content

Rhee C, Baker M, Vaidya V, et al; CDC Prevention Epicenters Program. Incidence of nosocomial COVID-19 in patients hospitalized at a large US academic medical center. *JAMA Netw Open*. 2020;3(9):e2020498. doi:10.1001/jamanetworkopen.2020.20498

**eTable 1.** Summary and Attribution of Infection Acquisition for Late-Onset COVID-19 Cases in Hospitalized Patients

**eTable 2.** Summary and Attribution of Infection Acquisition for COVID-19 Cases Diagnosed Within 14 Days of Hospital Discharge

This supplementary material has been provided by the authors to give readers additional information about their work.

© 2020 Rhee C et al. *JAMA Network Open.*

## eTable 1. Summary and Attribution of Infection Acquisition for Late-Onset Covid-19 Cases in Hospitalized Patients

| Case | Case Summary and Any Relevant Epidemiologic Factors | Assessment |
|---|---|---|
| 1 | Elderly patient with asthma and mitral regurgitation, who presented from home with 2 weeks of fevers, sweats, fatigue, rhinorrhea, headache, cough, and worsening dyspnea. Chest CT showed bilateral ground-glass opacities concerning for Covid-19. Admitted to ICU with high oxygen requirements. Initial nasopharyngeal RT-PCR test negative, but repeat RT-PCR test on hospital day 3 positive. | **Definitely Community-Acquired** (patient presented with signs/symptoms classic for Covid-19; initial false negative PCR test possibly due to long interval between symptom onset and presentation) |
| 2 | Elderly patient with obstructive sleep apnea who presented from home with fatigue and pancytopenia, admitted to oncology ward and found to have B-cell acute lymphoblastic leukemia. On hospital day 15, developed malaise, sore throat, cough, and febrile neutropenia; nasopharyngeal RT-PCR test positive. *Relevant epidemiologic factor: patient's wife had been visiting daily and developed fever and cough and tested positive for SARS-CoV-2 on hospital day 13.* | **Definitely Hospital-Acquired** (symptoms developed >14 days from hospitalization; source likely patient's wife who was visiting and had confirmed Covid-19 before patient developed symptoms) |
| 3 | Elderly patient with diabetes mellitus and hypertension who presented from home with dyspnea, abdominal discomfort, and altered mental status. Found to have diabetic ketoacidosis and acute cholecystitis. Not tested for SARS-CoV-2 initially. Admitted to ICU and treated with percutaneous cholecystostomy tube and insulin. Course complicated by prolonged delirium and agitation. On hospital day 10, became acutely hypoxemic, febrile, hypotension, and confused requiring emergent intubation. Nasopharyngeal RT-PCR test positive on hospital day 11. | **Likely Community-Acquired** (presented with symptoms consistent with Covid-19 but was not tested initially as another diagnosis was identified) |
| 4 | Young patient with lymphoma who was hospitalized for first cycle of chemotherapy, discharged home after 6 days. Re-presented from home on post-hospitalization day 6 for febrile neutropenia and typhlitis. Initial nasopharyngeal RT-PCR test negative on hospital days 2 and 4. Fevers resolved with antibiotics. On hospital day 8, developed new fevers and cough, nasopharyngeal RT-PCR test positive. *Relevant epidemiologic factor: patient lives with mother who was hospitalized with confirmed Covid-19 prior to patient's positive RT-PCR test.* | **Likely Community-Acquired** (patient likely acquired infection from mother prior to admission and virus was incubating when initially tested during hospitalization) |
| 5 | Middle-aged patient with relapsed leukemia following a stem cell transplant, admitted to oncology ward from home for salvage chemotherapy. On hospital day 3 developed febrile neutropenia with a new cough. First nasopharyngeal RT-PCR test sent on hospital day 4 positive. | **Likely Community-Acquired** (onset of symptoms on hospital day 3 suggests community acquisition) |
| 6 | Elderly patient with hypertension who presented from home with 2 weeks of fevers, gastrointestinal symptoms, chest pain, myalgias, and anosmia. Chest CT on admission showed multifocal ground-glass opacities. Labs consistent with inflammation. Initial nasopharyngeal RT-PCR tests on hospital days 1 and 2 were negative, but sputum sample sent on hospital day 4 returned positive. Serological tests (IgM and IgG) also positive. | **Definitely Community-Acquired** (presented with classic Covid-19 symptoms; initial negative tests likely due to long interval between symptom onset and presentation) |
| 7 | Middle-aged patient with rheumatic heart disease and tachy-brady syndrome who presented from home with several days of palpitations and nausea. On hospital day 2 developed hypoxia, fever, and myalgias. Chest CT showed ground-glass opacities. Nasopharyngeal RT-PCR test positive on hospital day 3. | **Definitely Community-Acquired** (onset of symptoms on hospital day 2) |
| 8 | Young patient with perinatally acquired human immunodeficiency virus with low CD4 count, history of stroke with residual weakness, who presented from home with several weeks of worsening mental status and neurologic deficits. MRI consistent with progressive multifocal leukoencephalopathy. Cerebrospinal fluid studies positive for JC virus. Had intermittent tachycardia since admission then developed fevers, | **Likely Community-Acquired** (tachycardia since admission likely sign of Covid-19; full-blown symptoms by day 6) |

© 2020 Rhee C et al. *JAMA Network Open.*

| | | |
|---|---|---|
| | tachypnea, and worsening tachycardia on hospital day 6. Nasopharyngeal RT-PCR test positive on hospital day 7. | |
| **9** | Elderly patient with diabetes mellitus who presented from home with 3 days of malaise, fatigue, dyspnea on exertion, cough, and nausea. Chest radiograph on admission showed bilateral opacities. Initial nasopharyngeal RT-PCR test negative x 2, but sputum sample sent on hospital day 7 returned positive. Serologies (IgG and IgM) sent on hospital day 3 were also positive. *Relevant epidemiologic factor: relatives at home had confirmed Covid-19.* | **Definitely Community-Acquired** (presented with classic Covid-19 symptoms; positive serologies on day 3 indicate infection acquired prior to admission) |
| **10** | Elderly patient with diabetes mellitus and congestive heart failure who presented from a nursing facility with a fall and femur fracture. Initial screening nasopharyngeal RT-PCR test negative. Underwent open reduction internal fixation of femur in the operating room on hospital day 3. Postoperatively became febrile and developed hypoxia, which was attributed to a possible urinary tract infection. Fevers resolved but course complicated by worsening renal failure. Screening nasopharyngeal RT-PCR test prior to discharge to rehabilitation facility on hospital day 7 returned positive. *Relevant epidemiologic factor: patient came from nursing facility during a period when Covid-19 outbreaks were common in long-term care facilities.* | **Likely Community-Acquired** (likely acquired Covid-19 at nursing facility; developed symptoms potentially consistent with Covid-19 on day 3) |
| **11** | Middle-aged homeless patient with hypertension and alcohol abuse, who presented intoxicated after a fall and developed seizures in the Emergency Department. Initial screening nasopharyngeal RT-PCR test negative. On hospital day 3, developed fevers and tachycardia. Repeat nasopharyngeal RT-PCR test positive. | **Likely Community-Acquired** (high risk for Covid-19 due to homelessness; symptom onset on day 3 suggests acquired prior to admission) |
| **12** | Middle-aged patient with diabetes mellitus and coronary artery disease who presented from home to an outside hospital with acute chest pain, dyspnea, nausea, and found to have a non-ST-elevation myocardial infarction. Initial screening nasopharyngeal RT-PCR test negative. Transferred to Brigham and Women's Hospital on hospital day 4. Repeat nasopharyngeal RT-PCR test negative. Underwent cardiac catheterization that showed stenosis of prior stents. Underwent chest CT on day 6 in preparation for cardiac surgery that showed patchy ground-glass opacities in setting of new cough, prompting a nasopharyngeal RT-PCR test on day 7 that was positive. *Relevant epidemiologic factor: patient works with homeless persons and was doing so up until day of admission.* | **Likely Community-Acquired** (patient was working with homeless persons until day of admission; non-ST-elevation myocardial infarction may have been symptom of Covid-19; ground-glass opacities on chest CT on day 6 suggest infection developing for days) |

© 2020 Rhee C et al. *JAMA Network Open.*

## eTable 2. Summary and Attribution of Infection Acquisition for Covid-19 Cases Diagnosed Within 14 days of Hospital Discharge

| Case | Case Summary and Any Relevant Epidemiologic Factors | Assessment |
|---|---|---|
| **13** | Young homeless patient who was admitted for 2 days under observation status for suicidal ideation. Not tested for SARS-CoV-2 on that index hospitalization. Re-presented 1 day after discharge requesting testing for sexually transmitted diseases after a sexual encounter with a man 3 days prior. Nasopharyngeal RT-PCR test positive. *Relevant epidemiologic factors: per patient, man with whom patient had a sexual encounter had symptoms of Covid-19. Also, local prevalence of Covid-19 high in homeless population.* | **Likely Community-Acquired** (brief index hospitalization; likely acquired from recent sexual contact with suspected Covid-19 case) |
| **14** | Middle aged patient with esophageal cancer admitted from home for 4 days for esophageal surgery. Not tested for SARS-CoV-2 during that index hospitalization. After discharge was doing well but underwent nasopharyngeal RT-PCR testing 2 days after discharge in light of his wife testing positive one week prior; patient tested positive. *Relevant epidemiologic factor: wife tested positive for SARS-CoV-2 1 week prior. Also exposed to a staff member who tested positive for SARS-CoV-2 (occurred after universal masking) .* | **Likely Community-Acquired** (brief index hospitalization; potentially acquired from staff member but more likely acquired from wife with confirmed Covid-19 one week prior given more sustained close contact with wife, and staff member was masked) |
| **15** | Elderly patient with idiopathic pulmonary fibrosis, lung transplant, and metastatic lung cancer, who presented from home and was hospitalized for 12 days with fever, cough, and dyspnea, felt to be secondary to fluid overload given marginal improvement with diuresis and negative cultures. Not tested for SARS-CoV-2 during that index hospitalization. Readmitted 3 days after discharge with worsening hypoxemia and altered mental status and bilateral infiltrates on chest radiograph. Nasopharyngeal swab weakly positive by RT-PCR, repeat swabs negative. | **Delayed Diagnosis** (Covid-19 was likely responsible for index hospitalization, but not tested as this occurred early in pandemic prior to aggressive testing practices) |
| **16** | Elderly patient with diabetes mellitus, chronic obstructive lung disease, esophageal cancer who presented from home with fatigue, cough, dyspnea, hypoxemia, and diarrhea for several weeks. Chest CT showed a right-sided pleural effusion. Underwent thoracentesis with improvement. Nasopharyngeal RT-PCR test negative x 2. Discharged home after a 5-day index hospitalization. 2 days later developed worsening dyspnea, cough, and diarrhea and re-presented to the Emergency Department. RT-PCR from nasopharyngeal swab weakly positive with borderline cycle threshold value. | **Delayed Diagnosis** (potentially acquired while in Covid ICU; alternatively, Covid-19 was responsible for index hospitalization and negative RT-PCR tests were false negatives given long duration of symptoms [supported by borderline cycle threshold PCR value on re-representation with similar ongoing symptoms]) |
| **17** | Elderly patient with cardiac disease and invasive urothelial carcinoma admitted from home for radical cystectomy, with complicated post-operative course including stroke, aspiration pneumonia, and arrhythmia requiring pacemaker. Discharged home after 16 day index hospitalization. 4 days later developed fevers, vomiting, and fatigue. Represented to the Emergency Department at post-hospitalization day 5 and found to be RT-PCR positive by nasopharyngeal swab. No known sick contacts since hospital discharge. | **Likely Hospital-Acquired** (long index hospitalization, short period of time at home, no sick contacts at home) |
| **18** | Elderly patient with significant cardiac history who was admitted from home with recurrent ventricular tachycardia in setting of 3 weeks of fatigue, malaise, and chest tightness without fever, dyspnea, or upper respiratory symptoms. Underwent cardiac catheterization and ventricular tachycardia ablation. Not tested for SARS-CoV-2 during index hospitalization and discharged home after 6 days. Then developed worsening chest pain, dizziness, dyspnea, fatigue, cough, and sore throat, represented to the Emergency Department on post-hospitalization day 6 and tested positive for SARs-CoV-2 by NP swab. | **Delayed Diagnosis** (Covid-19 may have been responsible for index hospitalization, but not tested as this occurred early in pandemic prior to aggressive testing practices) |

© 2020 Rhee C et al. *JAMA Network Open.*

| | | |
|---|---|---|
| **19** | Middle-aged patient with congenital heart disease who presented from home with severe heart failure exacerbation and non-ST-elevation myocardial infarction. Required cardiac bypass surgery and hemodynamic support and course complicated by renal failure. Multiple RT-PCR tests sent during hospitalization due to various respiratory symptoms and fevers, all negative. Discharged to rehabilitation facility after 6 week index hospitalization. 9 days later developed new cough in setting of known Covid-19 outbreak at the facility. Tested positive by nasopharyngeal RT-PCR test on post-hospitalization day 11. *Relevant epidemiologic factor: rehabilitation facility with Covid-19 outbreak.* | **Likely Community-Acquired** (diagnosed on post-hospitalization day 11, known Covid-19 outbreak at facility) |
| **20** | Elderly patient with hypertension who presented from home with a right hip fracture and underwent a hip hemiarthroplasty with uncomplicated post-operative course. Discharged to rehabilitation facility after 5 day index hospitalization. On post-discharge day 11, developed fevers in context of several days of cough and sore throat, with known Covid-19 outbreak at the facility. Tested positive by nasopharyngeal RT-PCR on post-hospitalization day 12. *Relevant epidemiologic factor: rehabilitation facility with Covid-19 outbreak.* | **Likely Community-Acquired** (diagnosed on post-hospitalization day 12, known Covid-19 outbreak at facility) |
| **21** | Elderly patient with gastric cancer who was admitted from home with abdominal pain and ascites and underwent paracentesis with relief. Stated on checkpoint inhibitor immunotherapy and discharged to skilled nursing facility after 11 day index hospitalization. The facility subsequent had numerous residents test newly positive for SARS-CoV-2. Patient re-presented to the Emergency Department on post-discharge day 12 with worsening abdominal pain and nausea/vomiting and new cough. Nasopharyngeal RT-PCR test positive. *Relevant epidemiologic factor: rehabilitation facility with Covid-19 outbreak.* | **Likely Community-Acquired** (diagnosed on post-hospitalization day 12, known Covid-19 outbreak at facility) |
| **22** | Elderly patient with metastatic colon cancer admitted from home with recurrent falls and then discharged to rehabilitation facility after 4 day index hospitalization. On post-discharge day 6 developed fever and dry cough; multiple other residents at facility tested newly positive for SARS-CoV-2. Sent to Emergency Department on post-dicharge day 13 and found to have renal failure and elevated inflammatory markers. Nasopharyngeal RT-PCR test positive. *Relevant epidemiologic factor: rehabilitation facility with Covid-19 outbreak.* | **Likely Community-Acquired** (diagnosed on post-hospitalization day 13, known Covid-19 outbreak at facility) |
| **23** | **Note: This is the same case as Patient 4 in Supplemental Table 1.** Young patient with lymphoma who was hospitalized for first cycle of chemotherapy, discharged home after 6 days. Re-presentedfrom home on post-hospitalization day 6 for febrile neutropenia and typhlitis. Initial nasopharyngeal RT-PCR test negative on hospital days 2 and 4. Fevers resolved with antibiotics. On hospital day 8, developed new fevers and cough, nasopharyngeal RT-PCR test positive. *Relevant epidemiologic factor: patient lives with mother who was hospitalized with confirmed Covid-19 prior to patient's positive RT-PCR test.* | **Likely Community-Acquired** (patient likely acquired infection from mother prior to admission and virus was incubating when initially tested during hospitalization) |

© 2020 Rhee C et al. *JAMA Network Open.*
